# Carotenoid-dependent singlet oxygen photogeneration in light-harvesting complex 2 of *Ectothiorhodospira haloalkaliphila* leads to the formation of organic hydroperoxides and damage to both pigments and protein matrix

**DOI:** 10.7717/peerj.16615

**Published:** 2024-01-16

**Authors:** Denis Yanykin, Mark Paskhin, Aleksandr Aleksandrovich Ashikhmin, Maxim Alexandrovich Bolshakov

**Affiliations:** 1 Institute of Basic Biological Problems, FRC PSCBR, Pushchino, Moscow, Russia; 2 Prokhorov General Physics Institute, Russian Academy of Sciences, Moscow, Moscow, Russia

**Keywords:** Purple sulfur bacteria, Carotenoids, Bacteriochlorophyll, Reactive oxygen species, Organic hydroperoxides, Light-harvesting complex 2

## Abstract

Earlier, it was suggested that carotenoids in light-harvesting complexes 2 (LH2) can generate singlet oxygen, further oxidizing bacteriochlorophyll to 3-acetyl-chlorophyll. In the present work, it was found that illumination of isolated LH2 preparations of purple sulfur bacterium *Ectothiorhodospira haloalkaliphila* with light in the carotenoid absorption region leads to the photoconsumption of molecular oxygen, which is accompanied by the formation of hydroperoxides of organic molecules in the complexes. Photoformation of two types of organic hydroperoxides were revealed: highly lipophilic (12 molecules per one LH2) and relatively hydrophobic (68 per one LH2). It has been shown that illumination leads to damage to light-harvesting complexes. On the one hand, photobleaching of bacteriochlorophyll and a decrease in its fluorescence intensity are observed. On the other hand, the photoinduced increase in the hydrodynamic radius of the complexes, the reduction in their thermal stability, and the change in fluorescence intensity indicate conformational changes occurring in the protein molecules of the LH2 preparations. Inhibition of the processes described above upon the addition of singlet oxygen quenchers (L-histidine, Trolox, sodium L-ascorbate) may support the hypothesis that carotenoids in LH2 preparations are capable of generating singlet oxygen, which, in turn, damage to protein molecules.

## Introduction

Photosynthesis is a key process that enables the existence of life on Earth, converts solar energy into the energy of organic substances, and drives oxygen evolution. Organic matter and oxygen are then used by most of the living organisms. Despite the undoubted importance of studying this process, the molecular mechanisms of photoreactions in the photosynthetic apparatus (PA) remain one of the unsolved problems in the study of photosynthesis.

Purple photosynthetic bacteria contain one of the most straightforward systems for collecting solar energy compared to similar systems in other photosynthetic organisms (algae, plants, *etc.*). It consists of three types of pigment-protein complexes: two antenna light-harvesting ones—LH1 and LH2, as well as reaction centers (RC). The antenna complexes are built according to the universal modular principle. The structural element of the complexes is α/β-heterodimer, which binds three bacteriochlorophyll (BChl) molecules and one carotenoid molecule in the LH2 complex, and 2 BChl molecules and one carotenoid molecule in the LH1 complex (Koepke et al., 1996; Papiz et al., 2003). In purple bacteria, LH2 complexes usually consist of 8–9 or 12–13 pairs of heterodimers (Papiz et al., 2003; Löhner et al., 2015; Leiger et al., 2019; Koepke et al., 1996; Qian et al., 2021; Qian et al., 2022). In the structure of the LH2 complex, BChl molecules form two rings, which are designated by the absorption bands of BChl at 800 and 850 nm in the near IR region as BChl800 and BChl850, respectively. Carotenoids are located between α/β-polypeptides, in the so-called “carotenoid pockets” and interact both with the amino acid residues of both polypeptides and with BChl molecules (Koepke et al., 1996; Papiz et al., 2003; Leiger et al., 2019).

In the cell of purple bacteria, carotenoids perform the following functions: (1) They extend the spectral range of absorbed solar energy in the blue–green region of the spectrum (430-570 nm), where BChl has a very low optical absorption, and then transfer the absorbed energy (in the form of excitation energy) to BChl molecules and further to RC (Koepke et al., 1996; Kereïche et al., 2008; Leiger et al., 2019); (2) They quench the excited triplet states of BChl, preventing the formation of reactive oxygen species (ROS) and protecting the antenna complex from oxidation, and they can also neutralize singlet oxygen, which is the most potent oxidizing agent, the formation of which leads to the oxidation of BChl, lipids, proteins and, ultimately, cell death (Hashimoto, Uragami & Cogdell, 2016). Finally, an equally important function of carotenoids is maintaining the structure of complexes due to hydrophobic interactions between carotenoids, polypeptides, and BChl (Moskalenko & Karapetyan, 1996; Cogdell, Gall & Köhler, 2006).

ROS can oxidize a variety of targets in the cell, including proteins, pigments, and lipids among other molecules (Gill & Tuteja, 2010). In photosynthetic organisms, ROS are formed, and then oxygen molecule receives energy from an excited photosensitizer molecule or by the one-electron reduction of oxygen in the photosynthetic electron-transfer chain (Pospíšil, 2012; Kreslavski et al., 2012; Schmitt et al., 2014; Arellano et al., 2007). In the case of LH2 of purple bacteria, such a photosensitizer is generally considered to be BChl, which in some cases can change from a singlet state to a long-lived triplet state and interact with oxygen to form singlet oxygen or other ROS (this type of reaction is inefficient and the yield in this reaction is relatively low) (Foote, 1977; Foote, 1979; Cogdell, Gall & Köhler, 2006; Telfer, Pascal & Gall, 2008; Frank & Polívka, 2009). The task of the cell is to prevent the formation of singlet oxygen by quenching the triplet state of BChl or quenching singlet oxygen. In both cases, the primary role is played by carotenoids capable of quenching ROS with heat dissipation. Such ideas about carotenoids’ role are generally accepted (Hashimoto, Uragami & Cogdell, 2016).

Previously, it has been shown that illumination of LH2 preparations in the region of absorption of carotenoids (in contrast to red light) induces the generation of singlet oxygen and the oxidation of BChl mediated by it to 3-acetyl-chlorophyll (Makhneva et al., 2019; Makhneva et al., 2020; Makhneva, Bolshakov & Moskalenko, 2021; Makhneva & Moskalenko, 2022). However, it is known that singlet oxygen can oxidize BChl and interact with protein molecules, damaging them (Wright et al., 2002; Davies, 2004). In this case, peroxide compounds are a product of this interaction. In this work, we investigated the possibility of the interaction of singlet oxygen formed upon illumination of LH2 preparations with the components of the proteins of the complex. Also, we determined one of the products of this interaction.

## Materials and Methods

Purple sulfur bacterium *Ectothiorhodospira haloalkaliphila* was grown on Pfenning’s medium under illumination of 2,000 lux at 26 ± 2 °C with a 75 W incandescent lamp (Imhoff & Trüper, 1977). LH2 preparations were prepared from cells of purple sulfur bacterium *Ectothiorhodospira haloalkaliphila* according to (Bol’shakov et al., 2016) and suspended (at 1.16 µM) in a medium containing 50 mM Tris-HCl (pH 7.5) and stored at −76 °C. The BChl concentration was determined in Tris-HCl solution (Klenina et al., 2022). PG200N Spectral PAR Meter (UPRtek, Zhunan, Taiwan) was used to estimate light spectra and flux density. The hydrodynamic diameter distribution of LH2 preparations was determined using Zetasizer Ultra (Malvern Panalytical, Malvern, UK) at 25 °C. Temperature dependence of viscosity of LH2 preparation in solution was obtained using SmartPave 102 rheometer (Anton Paar GmbH, Graz, Austria). The 3D fluorescence spectrum of LH2 preparations was measured using a Jasco FP-8300 spectrofluorimeter (JASCO Applied Sciences, Victoria, BC, Canada) at 25 °C. Absorption spectra were recorded on a Cary 50 spectrophotometer (Agilent Technology, Santa Clara, CA, USA). The temperature dependence of circular dichroism (CD) of LH2 complexes was measured on a Chirascan circular dichroism spectrometer (Applied Photophysics, Surrey, UK) with a thermostabilized cell, 1 mm cuvette, the optical density of samples at 850 nm was about 1; curve at 210 nm was plotted for samples normalized by protein concentration.

**Figure 1:**
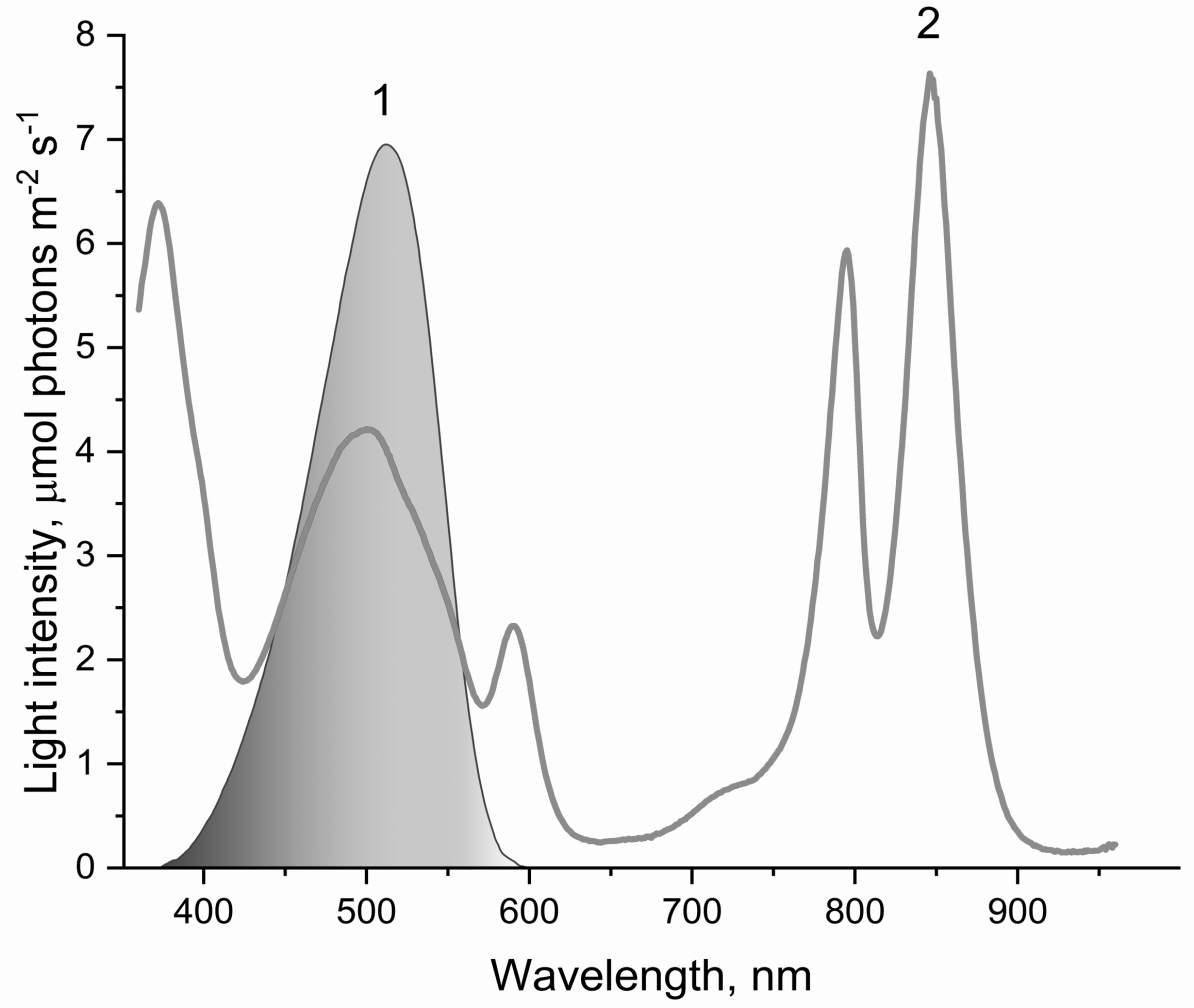
The spectrum of light used to illuminate LH2 preparations (1) and absorption spectrum of LH2 preparations (2). This light was obtained by passing the projector (LETI (LETI, Russia) with a 500 W KGM-220-500 lamp) light through a blue–green light filter (BGF-22 and YGF-12 (OOO Photooptic, Obninsk, Russia)). It should be noted that the data presented in curve 2 are demonstrative and not intended for estimating the quantum yield of photobleaching of pigments of the LH2 with statistical accuracy.

**Figure 2:**
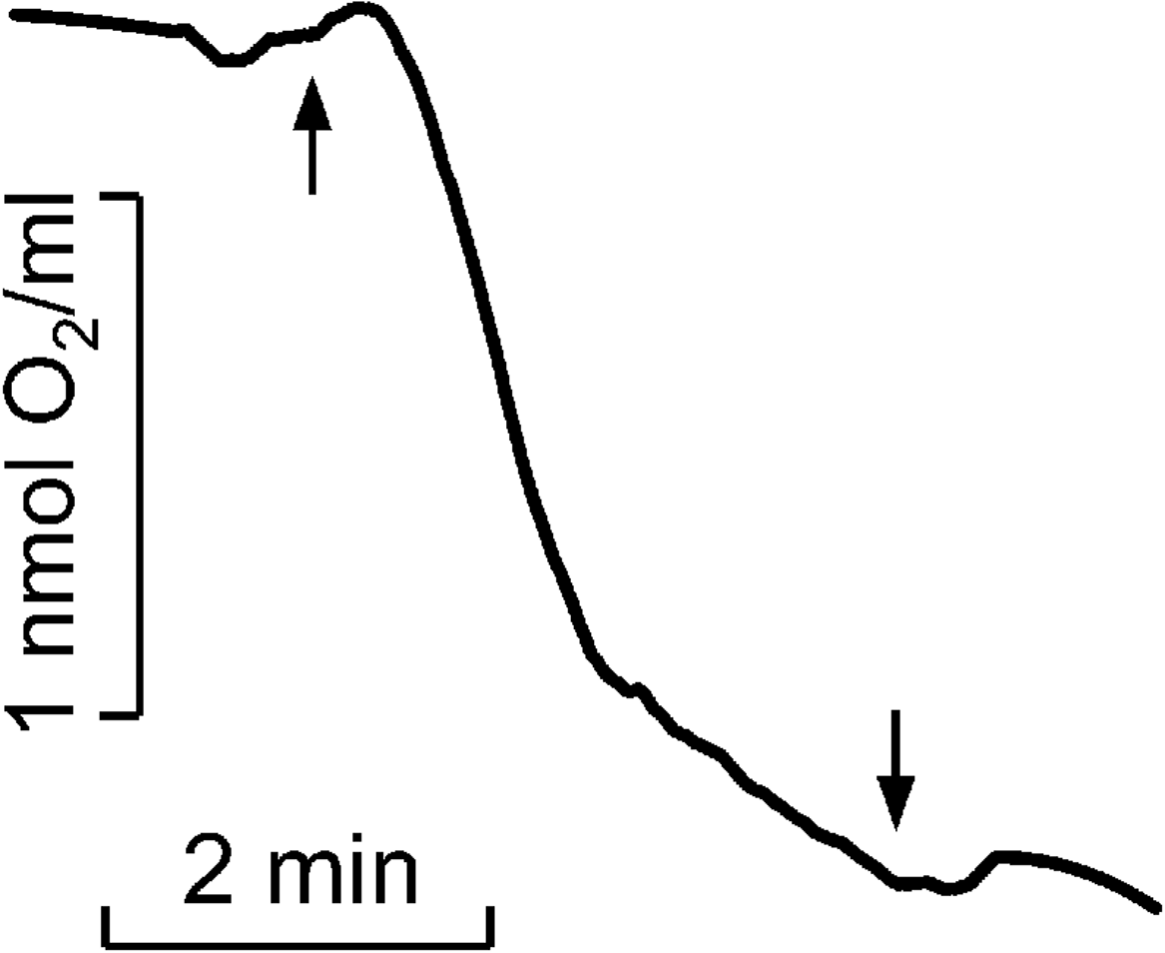
Kinetics of molecular oxygen photoconsumption in LH2 preparations. Measurements were carried out in the medium containing 50 mM Tris-HCl at LH2 concentration of 83.5 nmol at 25 °C. The measurements were done at least triplicate. For other details see “Materials and Methods”.

The oxygen photoconsumption rate in LH2 preparations was measured using a Clark-type electrode in DW2/2 Electrode Chamber (Hansatech Instruments Ltd., Norfolk, UK) under continuous illumination (375 > *λ* >600 nm (Fig. 1), 650 µmol photon s^−1^ m^−2^) at 25 °C and LH2 concentration of 83.5 nM. The approach described earlier was used to reveal the photoproduction of the organic hydroperoxides in LH2 preparations (Khorobrykh et al., 2011; Yanykin et al., 2017). Either preillumination (375 > *λ* >600 nm (Fig. 1) or *λ* >600 nm (Red filter KS11) (Table 1), 650 µmol photon s^−1^ m^−2^) or dark incubation (dark control) of the LH2 preparations was carried out for from five to 32 min in the medium containing 50 mM Tris-HCl at the LH2 preparations concentration of 16.7 nM at 25 °C. Figure 2 shows the < < light minus dark > > difference in the Spy-LHP fluorescence kinetics. This difference indicates the Spy-LHP oxidation by peroxides formed in LH2 preparations under illumination (Yanykin et al., 2017).

**Table 1:** Oxygen photoconsumption and photoproduction of R-OOH in LH2 preparations. HP-OOH are hydrophilic peroxides, LP-OOH are hydrophilic peroxides. His is L-histidine, AscNa is sodium L-ascorbate, RB is Rose Bengal, SOD is superoxide dismutase, Cat is catalase. The measurements were done at least triplicate. Standard deviation did not exceed 7%.

Sample	Oxygen photoconsumption, O_2_/(LH2 h)			Photoproduction of HP-OOH			Photoproduction of LP-OOH		
	During 1st min	After 1st min	Within 20 min, O_2_/LH2	µM	per one LH2	% by control	µM	per one LH2	% by control
LH2	993	165	69	0.11	68.39	100	2.05 10^−2^	12.25	100
LH2 (without illumination)^a^				0.003	1.60^*^	2	7.44 10^−5^	0.04^**^	0.4
LH2 (illuminated with light *λ*> 600 nm)				0.001	0.34^*^	0.5	−1.44 10^−4^	−0.09^**^	−0.7
LH2 + 10 mM His	590	436	148	0.58	348.8^*^	510	3.75 10^−3^	2.25^**^	18
LH2 + 20 mM trolox				0.26	152.9^*^	224	3.16 10^−3^	1.89^**^	15
LH2 + 100 mM AscNa				0.34	204.8^*^	300	−8.2 10^−4^	−0.49^**^	-4
16.7 nM RB + 10 mM His				0.25	152.7^*^	223	1.19 10^−3^	0.71^**^	6
16.7 nM RB + 20 mM trolox				1.52	912.3^*^	1334	5.46 10^−5^	0.03^**^	0.3
16.7 nM RB + 100 mM AscNa				0.46	277.8^*^	406	2.42 10^−4^	0.15^**^	1
LH2 + 200 un/ml SOD + 200 un/ml Cat				0.10	58.58	86	1.13 10^−2^	6.74^**^	55
LH2 + 200 un/ml SOD + 200 un/ml Cat (added after illumination)				0.11	66.34	97	2.11 10^−2^	12.62	103
LH2 +16.7 nM RB				0.12	72.86	107	3.88 10^−3^	2.32^**^	19
LH2 + 16.7 nM RB + 10 mM His				0.15	86.99^*^	127	6.95 10^−3^	4.16^**^	34
Pigment extract from LH2 (obtained after illumination of preparations)				9.14 10^−4^	0.55^*^	0.8	−2.45 10^−4^	−0.15^**^	−1.2

**Notes.**

* Statistically significant difference (*p* < 0:05) from control in the photoformation of HP-OOH.

** Statistically significant difference (*p* < 0:05) from control in the photoformation of LP-OOH.

a The amount of R-OOH in the preparations before and after of dark incubation (without illumination) was compared.

The intensity of Spy-LHP fluorescence quenching due to LH2 (at 16.7 nM LH2) was 8%. Exogenic redox agents used in this work did not affect the fluorescence of the Spy-LHP probe. Spy-LHP is a low-fluorescent compound that can be oxidized with hydroperoxides to form a high-fluorescent compound. According to the manufacturer’s description, Spy-LHP is highly specific for lipophilic organic hydroperoxides and does not react with hydroxyl radicals, superoxide anion, nitric oxides, peroxynitrite, and alkyl peroxyl radicals, and other species.

Two types of peroxide (highly lipophilic and relatively hydrophilic) had different reaction rates with Spy-HP. The highly lipophilic peroxide oxidized Spy-HP to the more fluorescent form SpyHPOx in 5 min. Still, this reaction occurred very slowly with hydrophilic peroxide and did not end after 180 min (Yanykin et al., 2017). The separation of pigments from the protein component of the complexes was carried out as follows. Pre-illuminated preparations or dark control (1 ml) were diluted in 9 ml acetone-methanol mixture (1:1) and shaken. A total of 4 ml of petroleum ether was added to the resulting solution and shaken again. After that, 10 ml of distilled water was added, and petroleum ether and pigments dissolved in it were taken with a pipette and evaporated in an argon flow. The pigments remaining in the vessel were washed from the remains of petroleum ether (it gave a side reaction with a fluorescent probe) and dissolved in 1 ml of ethanol. After that, 400 µl of the ethanol solution was added to 3,600 µl of the probe solution, and the fluorescence was measured as described above.

As previously described, the quantity of peroxides and highly lipophilic hydroperoxide was determined (Khorobrykh et al., 2011). The Spy165 LHP fluorescence kinetics of LH2 without MCPBA or TBHP was subtracted as background (Fig. 2, curve 4). All measurements were done at least three times, and corresponding average data are presented in Table 1 with standard deviations. In Fig. 2 the typical kinetics are shown.

## Results

It was shown that illumination of LH2 with light in the carotenoids absorption region led to molecular oxygen photo consumption at a rate of 993 µmol O_2_ (µmol LH2)^−1^ h^−1^ in the first minute of continuous illumination and 165 µmol O_2_ (µmol LH2)^−1^ h^−1^ later (Fig. 2). According to our previous studies (Makhneva et al., 2019; Klenina et al., 2022), the most probable process accompanying oxygen photoconsumption in LH2 preparations is the formation of singlet oxygen in LH2 followed by its interaction with BChl. However, taking into account the number of pigment molecules in LH2 and the calculated oxygen photoconsumption rate, we assumed that BChl and carotenoids are not the primary targets for singlet oxygen, and 3-acetyl-chlorophyll is not the main product of the interaction. It is widely known that the reaction of singlet oxygen with proteins results in the formation of long-lived peroxide species of amino acids (Davies, 2004; Wright et al., 2002).

**Figure 3:**
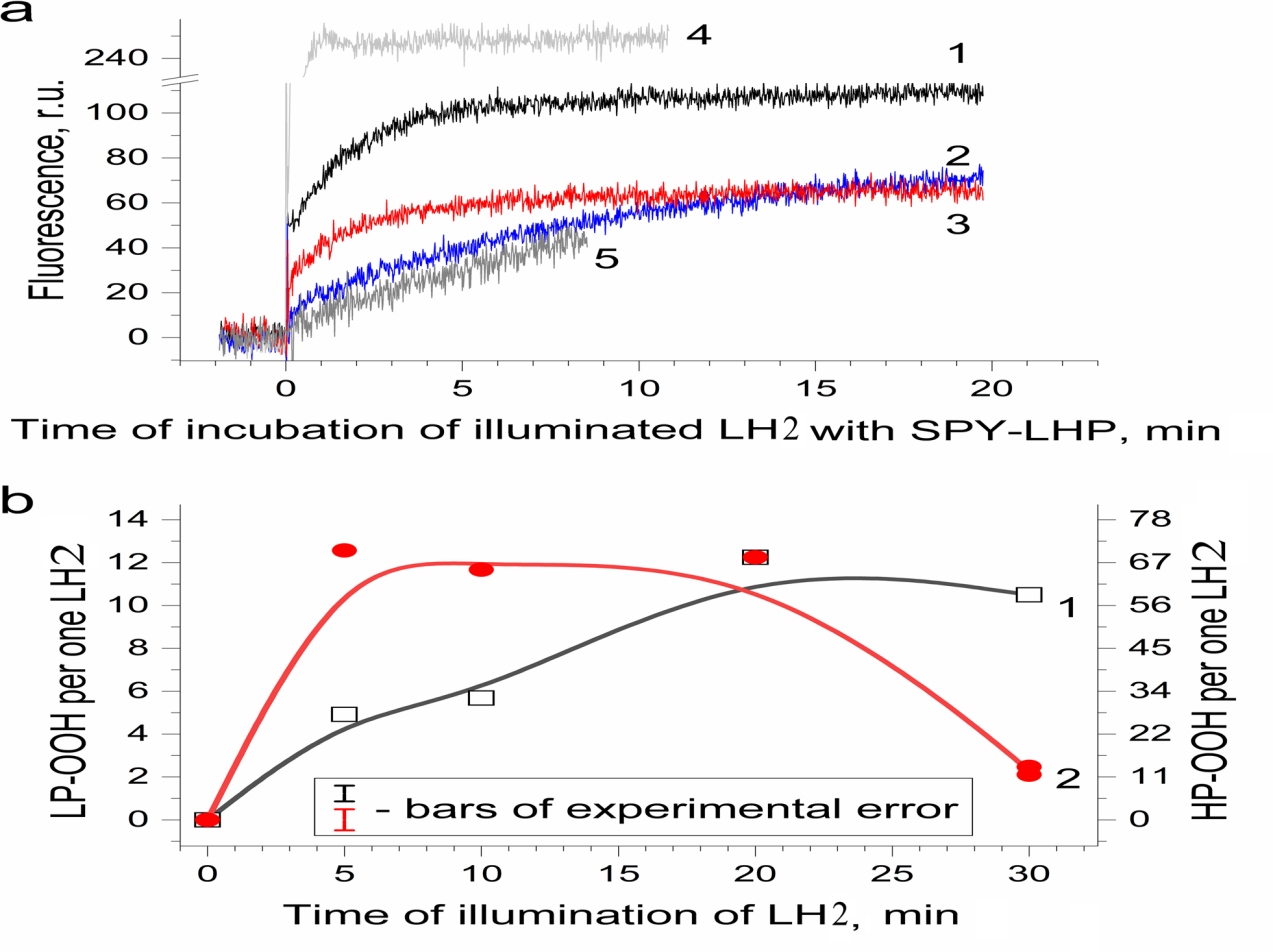
Photoproduction of R-OOH in LH2 preparations. (A) Kinetics of the Spy-LHP fluorescence related to its oxidation by peroxides photoproduced during a 20-min illumination of LH2 preparations in the absence (1) and in the presence of 10 mM L-histidine (2) or200 U/ml Catalase (3). Trace 4 is the kinetics of Spy-LHP fluorescence induced by the addition of 0.5 µM of MCPBA (a standard of lipophilic peroxide) used as a base for quantitative determination of LP-OOH. Trace 5 is the kinetics of Spy-LHP fluorescence induced by the addition of 1.67 µM of TBHP (a standard of hydrophilic peroxide) used as a base for quantitative determination of HP-OOH. (B) Dependence of LP-OOH (1) and HP-OOH (2) photoproduction on time of illumination of LH2 preparations. Either pre-illumination or dark incubation (dark control) of the LH2 preparations was carried out in the medium containing 50 mM Tris-HCl, at LH2 concentration of 16.7 nmol at 25 °C. Then an aliquot (400 µl) of the sample was added to 3,600 µl of 1.35 µM Spy-LHP dissolved in ethanol. Incubation of LH2 preparations with Spy-LHP was done at 37 °C. Incubation of MCPBA or TBHP with Spy-LHP was done in the presence of non-illuminated LH2 preparations at concentration of 1.67 nmol at 37 °C. The measurements were done at least triplicate. For other details see “Materials and Methods”.

Earlier, a fluorescence probe (Spy-LHP) specific to lipophilic peroxides has been used to detect hydroperoxides of organic molecules photoproduced in photosystem II preparations isolated from leaves of higher plants (Khorobrykh et al., 2011). Later, the probe was successfully used to detect organic hydroperoxides in samples isolated from photosynthetic organisms. The probe was applied to detect hydroperoxides in the present work. Figure 3A shows the difference in “light minus dark” kinetics of the fluorescence of the Spy-LHP related to its oxidation with peroxides photoformed due to a 20-min illumination (650 µmol photon s^−1^ m^−2^) of LH2 preparations with blue–green light (curve 1). The fluorescence kinetics consists of two phases. The first one was rapid and reflected the photoproduction of highly lipophilic hydroperoxides (LP-OOH) in LH2. At the same time, the second one (observed after 5 min of measurement) was a slow, constant increase in fluorescence intensity owing to the interaction of Spy-LHP with relatively hydrophilic organic hydroperoxides (HP-OOH). Thus, we demonstrated that illumination of the LH2 preparations can result in the photoproduction of LP-OOH (detected in the fast kinetics component) and HP-OOH (slow component).

Figure 3B shows the amount of formed hydroperoxides as a function of illumination time. The total amount of the hydroperoxides (R-OOH) increased with increasing illumination time. They reached the maximum level (up to 80 molecules per one LH2) at the 20th minute, and a decrease in R-OOH content accompanied further illumination. At the same time, LP-OOH was more stable than HP-OOH: the total amount of R-OOH decreased due to the decline in the amount of HP-OOH (compare curve 1 and curve 2). The maximum level of HP-OOH was observed from 5 to 20 min (68 molecules per one LH2), while the maximum level of LP-OOH (12 molecules per one LH2) was reached only after 20 min of illumination. Even though precursors of HP-OOH were relatively many, their photoproduction was saturated relatively quickly, indicating that the precursors of HPOOH were more available for oxidation. Despite their small amount, Precursors of LP-OOH are entirely converted into hydroperoxides in a relatively long time, which may indicate their relatively low availability for oxidation. Further studies were performed using preparations illuminated for 20 min since this illumination time provides the maximum photoproduction of both types of hydroperoxides. It was shown that the generation of R-OOH in LH2 was related to the sample illumination since incubation of LH2 preparation in the dark did not lead to the formation of organic hydroperoxides (Table 1). Moreover, illumination of the LH2 complexes with light, which is not absorbed by carotenoids (*λ* > 600 nm), did not lead to R-OOH photoproduction.

**Figure 4:**
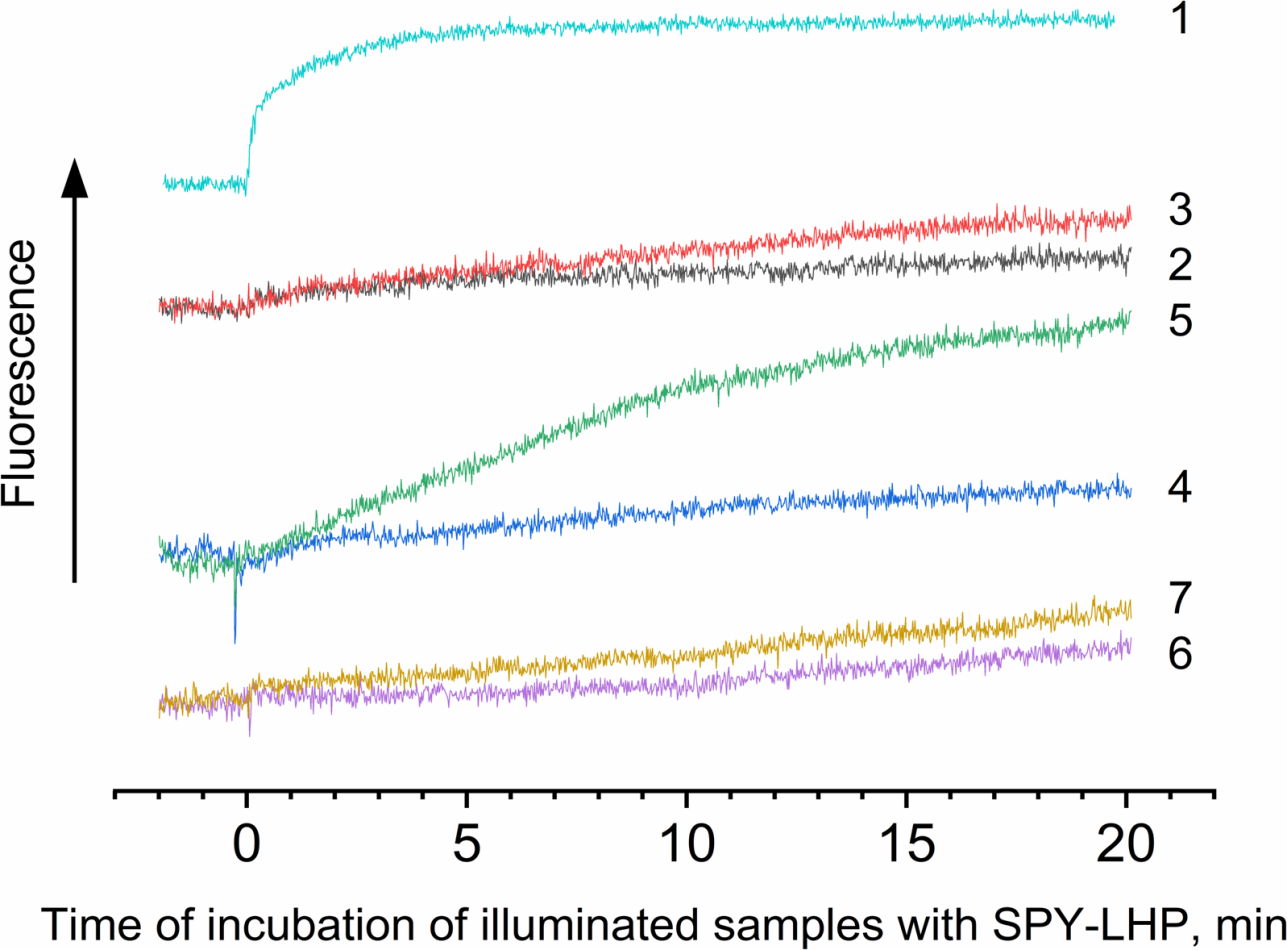
Photoproduction of R-OOH. Samples containing either LH2 preparations (1, 2, 4, 6) or Rose Bengal (3, 5, 7) as a photosensitizer in the absence (1) and in the presence of 10 mM L-histidine (2, 3) 20 mM trolox (4, 5), or 100 mM sodium L-ascorbate (6, 7). The measurements were done at least triplicate. For other details see Fig. 3 and “Materials and Methods”.

**Figure 5:**
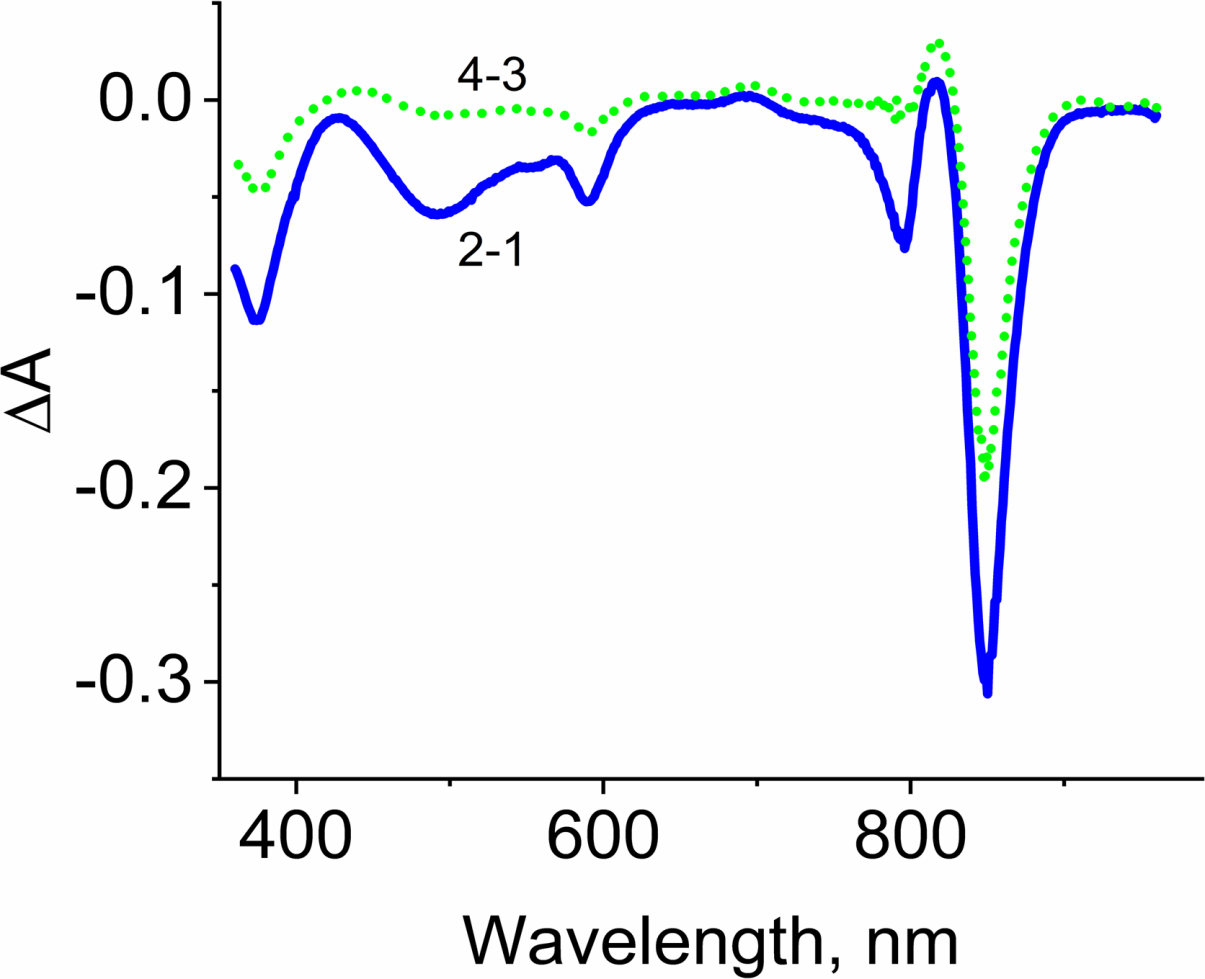
Difference (light minus dark) absorption spectra of LH2 preparations. Either pre-illumination or dark incubation (dark control) of the LH2 preparations was carried out in the absence (2-1) or in the presence (4-3) of 10 mM L-histidine in the medium containing 50 mM Tris-HCl, at LH2 concentration of 16. 7 nmol at 25 °C. Measurements of the absorption spectra were done immediately after pre-illumination or dark incubation without dilution of the sample solution.

It is known that interaction with protein molecules leading to the formation of peroxide compounds can be carried out by singlet oxygen and other ROS species (Schubert, Watson & Baecker, 1969). To confirm or disprove the involvement of singlet oxygen, superoxide anion radical, or hydrogen peroxide in the photoproduction of hydroperoxides in LH2, experiments were performed using singlet oxygen quenchers and superoxide dismutase and catalase. It was shown that the addition of 10 mM L-histidine led to, on the one hand, a drastic decrease in LP-OOH photoproduction (by 80%) and, on the other hand, an increase in the HP-OOH photoproduction (by factor 5) (Table 1). Note that the addition of L-histidine (or other adds) to the preparations did not lead to the production of R-OOH in the dark. Other singlet oxygen quenchers also decrease the LP-OOH photoproduction and increase HP-OOH photoproduction (Table 1 and Fig. 4, curves 2, 4, and 6). To test the hypothesis that the increase in the amount of HP-OOH in the presence of singlet oxygen quenchers may be due to the formation of oxidized quencher products (*e.g.*, histidine hydroperoxides), we investigated the photogeneration of quencher hydroperoxides in the presence of the 0.0167 µM Rose Bengal (RB) as singlet oxygen generating photosensitizer. It was shown that illumination of the solution containing the 10 mM L-histidine (or 20 mM Trolox, or 100 mM sodium L-ascorbate) and 0.0167 µM RB (in the absence of the LH2 preparations) led to photoformation of oxidized products, which can react with Spy-LHP like relatively hydrophobic hydroperoxides (Fig. 4, curves 3, 5 and 7). Surprisingly, illumination of LH2 preparations in the presence of an equimolar concentration of RB (in both the presence and absence of L-histidine) did not increase in the R-OOH photoproduction. Moreover, it was revealed the decrease in the photoformation of the LP-OOH (Table 1). Addition of SOD and catalase before illumination of LH2 practically did not lead to inhibition of HP-OOH photoproduction and decreased the LP-OOH photoproduction by factor two. The enzymes added after lighting the preparations did not affect R-OOH content (Table 1). The obtained data make it possible to exclude the participation of hydrogen peroxide and the superoxide anion radical in the formation of hydroperoxides. However, it cannot be excluded that SOD and catalase may have protective effects against damage (as sacrificial antioxidants), which could take place during illumination. The absence of photoformed hydroperoxides in the fraction containing pigments may indicate that protein molecules are the main targets for oxidation (Table 1). Oxygen photoconsumption leading to organic hydroperoxides photoformation is accompanied by changes in photoabsorption and photoluminescence properties of LH2 preparations. It was shown that illumination of LH2 preparations led to photobleaching of pigments of the complexes. In particular, strong photobleaching was observed in the BChl absorption bands (Fig. 5).

**Figure 6:**
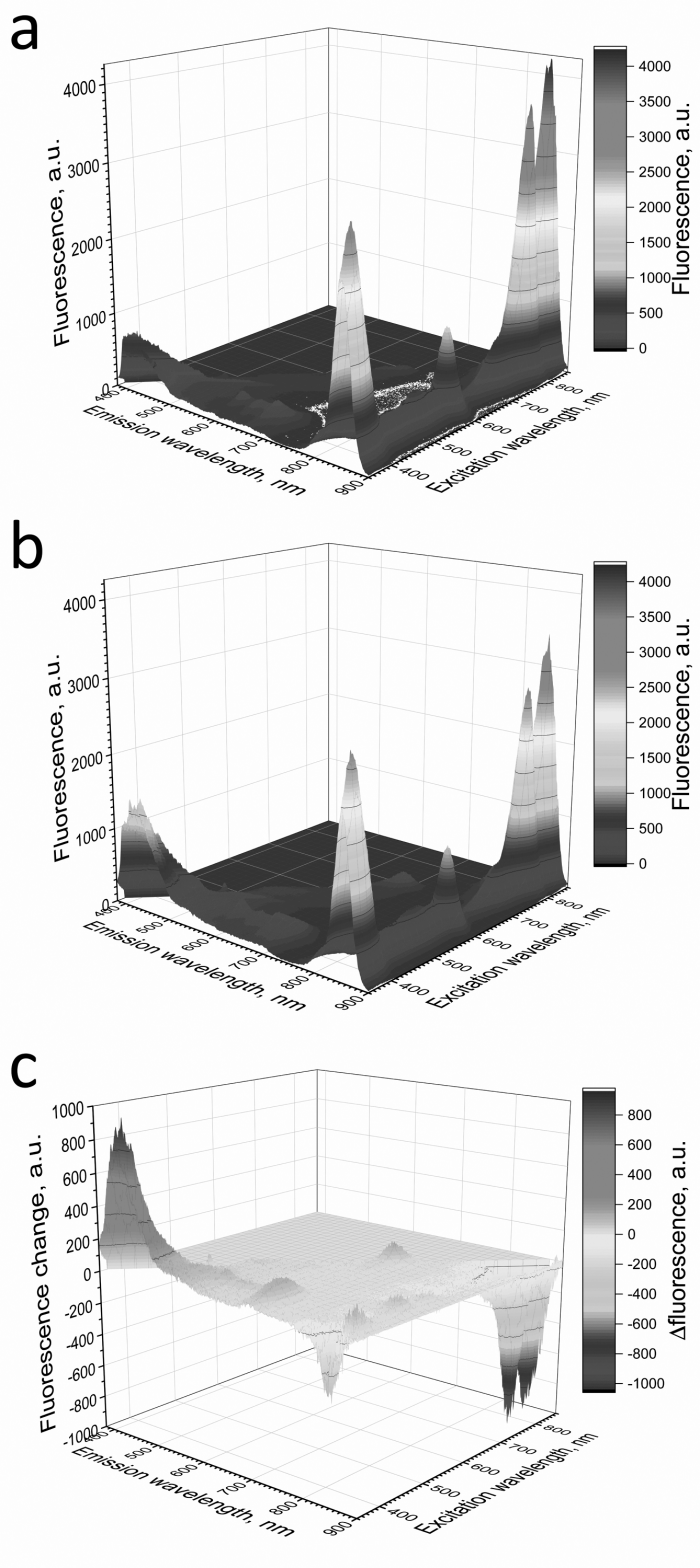
Fluorescence 3D spectra of LH2 preparations. Fluorescence spectra of LH2 preparations before (A) and after (B) illumination. (C) Shows a difference (light minus dark) fluorescence spectra of LH2 preparations. Either pre-illumination or dark incubation (dark control) of the LH2 preparations was carried out in the medium containing 50 mM Tris-HCl, at LH2 concentration of 16.7 nmol at 25 °C. Measurements of the fluorescence spectra were done immediately after pre-illumination or dark incubation without dilution of the sample solution.

**Figure 7:**
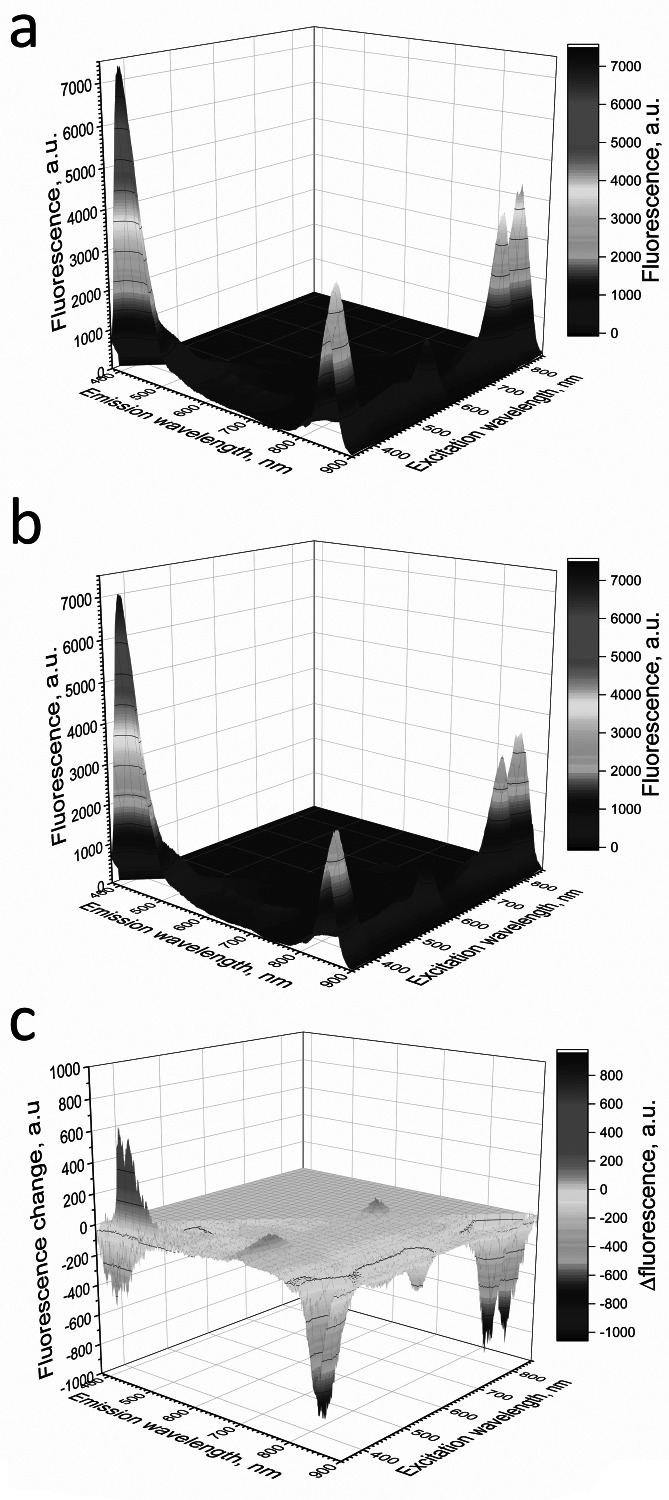
Fluorescence 3D spectra of LH2 preparations. LH2 preparations before (A) and after (B) illumination. (C) Shows difference (light minus dark) fluorescence spectra of LH2 preparations. Either pre-illumination or dark incubation (dark control) of the LH2 preparations was carried out in the medium containing 50 mM Tris-HCl and 10 mM L-histidine at LH2 concentration of 16.7 nmol at 25 °C. Measurements of the fluorescence spectra were done immediately after pre-illumination or dark incubation without dilution of the sample solution.

Adding L-histidine (or Trolox or sodium L-ascorbate) inhibited the photobleaching. Furthermore, the illumination of LH2 preparations led to changes in the fluorescence spectrum. It was shown that preillumination of LH2 preparation led to both a decrease in the BChl fluorescence intensity and an increase in the fluorescence of protein components of the complexes (Fig. 6), probably due to oxidation of the pigments and damage to protein molecules. The addition of L-histidine prevents both a decrease in the BChl fluorescence and an increase in the fluorescence of proteins (Fig. 7). Moreover, a reduction in L-histidine fluorescence was observed in the difference spectrum (Fig. 7A) that can be attributed to the oxidation of the amino acid and formation of its endoperoxide.

**Figure 8:**
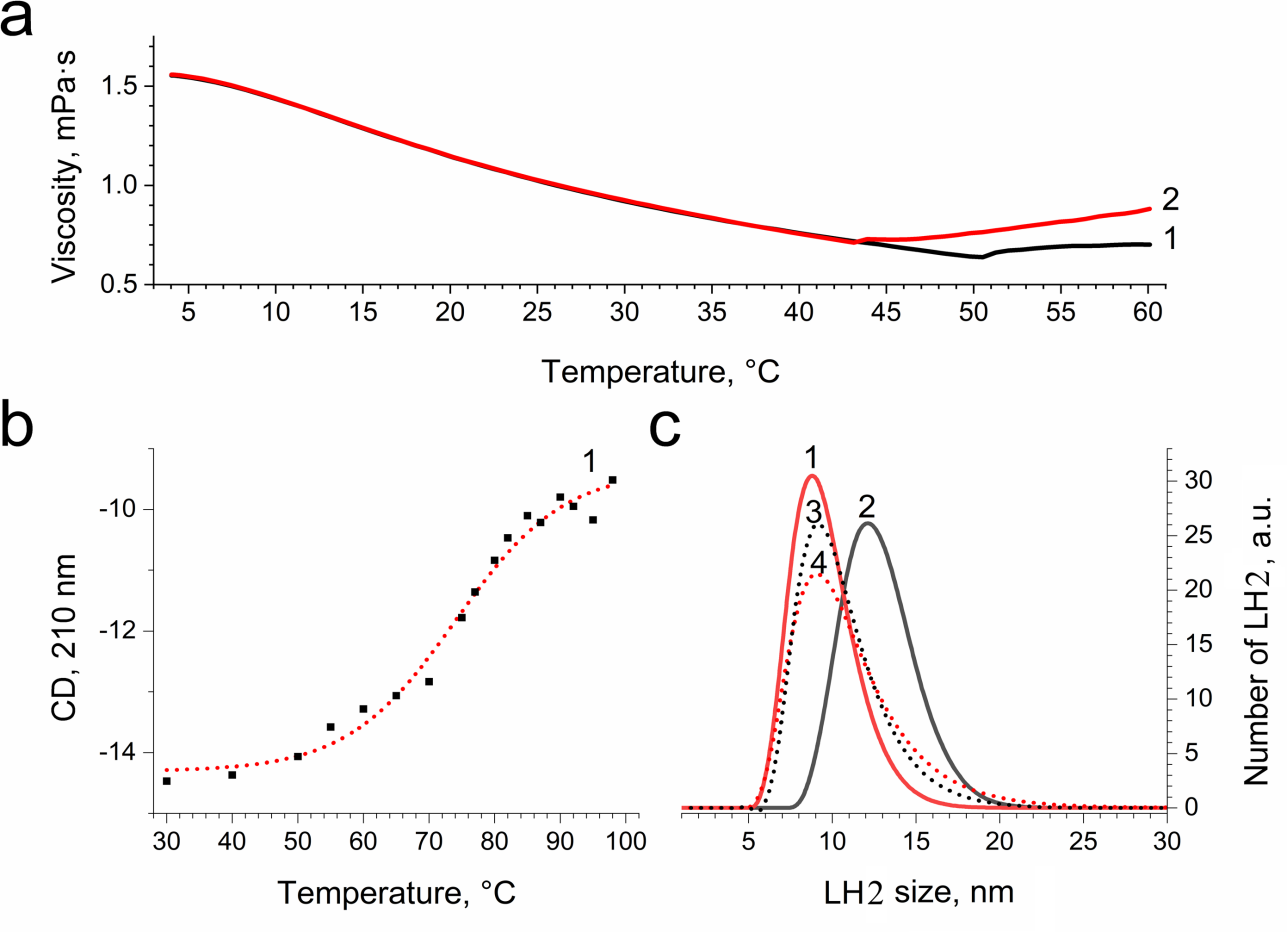
Dependence of viscosity (A) and CD signal at 210 nm (B) of LH2 solution on temperature and size distribution of LH2 obtained using dynamic light scattering. Measurements were performed before (1, 3) and after (2, 4) illumination with blue–green light in the absence (1, 2) and presence (2, 4) of 10 mM L-histidine. Either pre-illumination or dark incubation (dark control) of the LH2 preparations was carried out in the medium containing 50 mM Tris-HCl at LH2 concentration of 16.7 nmol at 25 °C. Measurements were done immediately after pre-illumination or dark incubation without dilution of the sample solution.

In order to confirm damage to proteins of the LH2, we investigated the temperature dependence of the sample viscosity before and after illumination, which may reflect light-induced changes in the stability of the complexes. Fig. 8A indicates that the heating of LH2 led to changes in the state of the preparations, which can reflect the destruction of the complex. The transition started at 50 °C (curve 1). These findings correlated well with CD data (Fig. 8B), confirming the changes begin at 50 °C. Preillumination of the LH2 preparations significantly shifts the transition point to lower temperatures (43 °C) (Fig. 8B, curve 2). Moreover, preillumination of the LH2 increases the hydrodynamic radius of the complexes (Fig. 8C, curves 1 and 2) but was not observed under the addition of 10 mM L-histidine (curves 3 and 4). Oxidative damage to protein molecules was proposed to increase the hydrodynamic radius of the complexes and modify endogenous fluorescence quencher that leads to change in fluorescence intensity (Bystranowska et al., 2012; Neves-Petersen et al., 2002; Correia et al., 2012a; Correia et al., 2015; Correia et al., 2012b; Correia et al., 2014). Thus, the presented data support the possibility that carotenoid-dependent singlet oxygen photogeneration in LH2 of *Ectothiorhodospira haloalkaliphila* leads to the formation of organic hydroperoxides and damage to both pigments and protein matrix.

## Discussion

It is known that the interaction of singlet oxygen with organic molecules (chemical quenching) results in the consumption of molecular oxygen from the measurement medium (Matheson et al., 1975; Weil, 1965). In our experiments, illumination of LH2 preparations led to oxygen uptake at a rate of up to a thousand µmol O_2_ (µmol LH2)^−1^ h^−1^ (at the beginning of illumination). In this case, the components of LH2 were both the photosensitizer and the chemical quencher. Note that the observed rates are very slow. However, even at such a low rate of oxygen photoconsumption, about 70 oxygen molecules were consumed per one LH2 complex during 20 min of illumination, comparable to the total amount of R-OOH (80/LH2) (Table 1). However, in addition to reactions leading to the formation of peroxide compounds, singlet oxygen can be consumed in other reactions not accompanied by R-OOH formation. In addition, the amount of photoconsumed oxygen in the presence of L-histidine was half that of organic peroxides. Therefore, the estimated amount of R-OOH is approximate, and the possible error may be due to the different properties of R-OOH and the model hydroperoxides used for calibration, as well as different concentrations of preparations in the measurements of the rate of oxygen photoconsumption and formation of hydroperoxides.

The data shows that the LP-OOH/HP-OOH ratio is 1/5.5 at 20-min of illumination. However, the LP-OOH/HP-OOH ratio changed during illumination. Differences in the availability of molecule precursors for oxidation and the stability of formed R-OOH can explain it. The amount of HP-OOH quickly reached the maximum level and sharply reduced after 20 min of illumination. At the same time, LP-OOH is shown to be more stable, and their precursors are relatively hardly oxidizable. Earlier, it has been demonstrated that peroxides photoformed in photosystem II preparations spontaneously decomposed or were scavenged by 50% in approximately 12 min (Khorobrykh et al., 2011). Results of calculations of the amount of the hydroperoxides photoformed in samples under their illumination in the presence of various additions are presented in Table 1. The table shows that the singlet oxygen quenchers significantly reduce the amount of LP-OOH formed due to the illumination of LH2 preparations. These data confirm the involvement of singlet oxygen in LPOOH production. However, quenchers increased the photoformation of HP-OOH. Moreover, the amount of HP-OOH has become several times more than in the absence of quenchers. We assume this effect may be due to the interaction of L-histidine and singlet oxygen reaction products with fluorescent probes. Our data obtained using RB (artificial source of singlet oxygen) (Fig. 4) and previous studies (Tomita, Irie & Ukita, 1969; Schubert, Watson & Baecker, 1969; Wei et al., 2007; Liu et al., 2014) confirm this assumption. Unexpected results have been obtained by illuminating LH2 preparations in the presence of RB. We expected that adding an additional source of singlet oxygen would significantly increase, or at least not change (in case all precursor molecules capable of being oxidized to R-OOH were oxidized in the absence of RB) the amount of organic hydroperoxides. However, RB added in the presence or absence of L-histidine did not substantially change the amount of HP-OOH compared to the control and strongly inhibited LP-OOH photogenesis. The nature of this phenomenon needs to be clarified and requires further research. In the presence of RB, the dominant “application” of singlet oxygen is the oxidation of BChl, carotenoids, or proteins without forming lipophilic hydroperoxides. Our experiments (Fig. S1) show an increase in photobleaching of pigments while the amount of LP-OOH decreases.

Inhibition of LP-OOH photoproduction is shown under the addition of SOD and catalase (known superoxide anion radical and hydrogen peroxide scavengers). At the same time, SOD and catalase are not capable of decreasing the HP-OOH formation. In the case of the generation of superoxide anion radical and hydrogen peroxide, the enzymes would inhibit the formation of both types of hydroperoxides with equal efficiency since these ROS would be quenched before they could react with organic molecules. In our case, SOD and catalase can act as a sacrificial protein that reacts with a significant part of the producing singlet oxygen. At the same time, the enzymes do not prevent the formation of HP-OOH due to the high reaction rate of their precursor molecules with the singlet oxygen. As mentioned above, the oxidation of LP-OOH precursor molecules takes more time, and SOD and catalase can effectively compete with LP-OOH precursors for singlet oxygen. Thus, the effect of enzymes may be due to different reaction rate constants of two groups of precursor molecules with singlet oxygen rather than the formation of superoxide anion radical and hydrogen peroxide. Singlet oxygen generation has previously been shown both in pigment solutions (Borland et al., 1987; Egorov & Krasnovsky Jr, 1991; Krasnovsky Jr et al., 1993; Hoebeke & Damoiseau, 2002) and in isolated antenna complexes of bacteria (Makhneva et al., 2019; Makhneva et al., 2020; Makhneva, Bolshakov & Moskalenko, 2021; Makhneva & Moskalenko, 2022; Klenina et al., 2019) and plants (Krieger-Liszkay, 2004; Kramer & Mathis, 1980; Santabarbara et al., 2002; Rinalducci, Pedersen & Zolla, 2004). Currently, the dominant point of view is that the primary and only source of singlet oxygen in the antenna complexes of photosynthetic bacteria is BChl (Arellano et al., 2007; Uchoa et al., 2008). According to another hypothesis, carotenoids in LH2 can generate singlet oxygen, which can oxidize the BChl to 3-acetyl-chlorophyll.

Foote with co-authors (Foote, Chang & Denny, 1970; Foote, 1979) estimated the relative energies of ^3^Car and singlet oxygen depending on the number of conjugated double bonds (N). At N ≥ 11, the triplet state of such carotenoids turns out to be higher than the ^1^O_2_* level, and the process of excitation of oxygen into the singlet state upon interaction with ^3^Car becomes energetically possible. The mechanism of singlet oxygen generation upon excitation of carotenoids is considered in earlier work (Klenina et al., 2022). Triplet states of carotenoids can be formed in the process of singlet-triplet excitation fission according to the equation: ^1^Car* + Car → ^3^Car + ^3^Car. This process is spin-allowed and is quite well-known in the photophysics research of organic compounds (Smith & Michl, 2010; Smith & Michl, 2013). It was previously described in the case of LH2 of *Alc. vinosum* (Klenina et al., 2019; Klenina et al., 2022; Gryaznov et al., 2019). The population of ^3^Car by excitation fission occurs exceptionally quickly, in the pico- and sub-picosecond time range (Gradinaru et al., 2001). The quantum yield of this process in another purple bacterium (*Rhodospirillum rubrum*) was estimated to be 0.32, which is a reasonably high value (Gradinaru et al., 2001). The LH2 complexes from *Ectothiorhodospira haloalkaliphila* used in this work contain the following carotenoids: anhydrorhodovibrin 37.1% (*N* = 12), spirilloxanthin 29.8% (*N* = 13), lycopene 17.2% (*N* = 11), rhodopin 13.5% (*N* = 11), didehydrorhodopine 2.2% (*N* = 11) (Razjivin et al., 2021). Thus, all carotenoids that are part of light-harvesting complexes can theoretically, in an excited state, transfer energy to triplet oxygen with the formation of its singlet state. Our results of illuminating LH2 complexes into the Qy BChl absorption band are consistent with those discussed above (Table 1). It was revealed that illumination of the samples in the absorption band of carotenoids is necessary for forming organic hydroperoxides.

Carotenoid molecules in bacterial LH2 are located in the membrane in such a way that they effectively interact with both BChl molecules and amino acid residues of polypeptides (Freer et al., 1996; Prince et al., 1997; Prince et al., 2003; Papiz et al., 2003; Gabrielsen, Gardiner & Cogdell, 2009; Löhner et al., 2015). Thus, the generation of singlet oxygen in the immediate vicinity of a carotenoid can lead to the oxidation of both BChl and proteins. The present work found that the photoproduction of ROOH is not associated with pigment molecules. We assume that, besides pigments, proteins are targets for singlet oxygen. Using plant LHC2, singlet oxygen may induce the degradation of light-harvesting proteins (Lindahl, Yang & Andersson, 1995; Zolla & Rinalducci, 2002). It has been shown that protein tyrosine, tryptophan, and histidine can be oxidized by singlet oxygen to form hydroperoxides (Nakagawa, Okajima & Hino, 1976; Saito et al., 1977; Tomita, Irie & Ukita, 1969; Kang & Foote, 2000; Wright et al., 2002; Davies, 2004; Giles, 2007; Liu et al., 2014; Wei et al., 2007; Nakane et al., 2022; Nakane et al., 2021) *via* stage of an unstable endoperoxide intermediate. The interaction of peroxide-containing proteins with other biomolecules can result in secondary damage. Peroxides generated on peptides and proteins by singlet oxygen are poorly repaired (Davies, 2004; Prestwich et al., 2005; Matheson et al., 1975). We have shown that changes in the structure and properties of the protein matrix of the complex accompany the illumination of LH2. On the one hand, because of illumination, an increase in the hydrodynamic radius, which indicates a less compact structure of illuminated LH2, and a decrease in the thermal stability of the complexes are observed. On the other hand, the fluorescence intensity in the protein region increases. It is known that damage to proteins (including as a result of oxidation by singlet oxygen) can be accompanied by both a decrease in fluorescence and its increase (Bystranowska et al., 2012; Neves-Petersen et al., 2002; Correia et al., 2012a; Correia et al., 2015; Correia et al., 2012b; Correia et al., 2014). The effect largely depends on which protein components are damaged. All of the above changes are entirely or partially prevented by adding a singlet oxygen quencher, which confirms the participation of singlet oxygen in the destructive processes in the proteins of LH2. In this work, we used the specific fluorescence probe to reveal that photogeneration of singlet oxygen in LH2 led to photodamage to protein molecules and photoproduction of organic hydroperoxides.

## Supplemental Information

10.7717/peerj.16615/supp-1 Supplemental Information 1 Kinetics of molecular oxygen photoconsumption in LH2 preparations (83.5 nM) from Ectothiorhodospira haloalkaliphila upon addition of histidine (10 mM) and illumination with blue light (375 >*λ*>600 nm, 650 µmol photon s −1 m −2)

10.7717/peerj.16615/supp-2 Supplemental Information 2 Calibration of photoconsumption of molecular oxygen using sodium dithionite at 25 °C LH2 preparations from Ectothiorhodospira haloalkaliphila.

10.7717/peerj.16615/supp-3 Supplemental Information 3 Kinetics of molecular oxygen photoconsumption in LH2 preparations (83.5 nM) from Ectothiorhodospira haloalkaliphila without the addition of histidine and under blue light illumination (375 >*λ*>600 nm, 650 µmol photon s −1 m −2)

10.7717/peerj.16615/supp-4 Supplemental Information 4 Raw data exported from the Clark-type electrode in DW2/2 Electrode Chamber (Hansatech Instruments Ltd., UK) under continuous illumination (375 >*λ*>600 nm, 650 µmol photon s −1 m −2) at 25 °C and LH2 concentration of 83.5 nM and 10 mM L-histidine

10.7717/peerj.16615/supp-5 Supplemental Information 5 Raw data exported from the Clark-type electrode in DW2/2 Electrode Chamber (Hansatech Instruments Ltd., UK) under continuous illumination (375 >*λ*>600 nm, 650 µmol photon s −1 m −2) at 25 °C and LH2 concentration of 83.5 nM and without histidine

10.7717/peerj.16615/supp-6 Supplemental Information 6 Raw data exported from the Clark-type electrode in DW2/2 Electrode Chamber (Hansatech Instruments Ltd., UK) at 25 °C with addition sodium dithionite

10.7717/peerj.16615/supp-7 Supplemental Information 7 Raw data exported from the Zetasizer Ultra (Malvern Panalytical, Malvern, UK) at 25 °C Hydrodynamic diameter distribution of LH2 preparations was measured before illumination with blue–green light (375 > *λ* >600 nm, 650 µmol photon s −1 m −2) in the absence of 10 mM L-histidine.

10.7717/peerj.16615/supp-8 Supplemental Information 8 Raw data exported from the Zetasizer Ultra (Malvern Panalytical, Malvern, UK) at 25  °C Hydrodynamic diameter distribution of LH2 preparations was measured after illumination with blue–green light (375 > *λ* >600 nm, 650 µmol photon s −1 m −2) with 10 mM L-histidine.

10.7717/peerj.16615/supp-9 Supplemental Information 9 Raw data exported from the Zetasizer Ultra (Malvern Panalytical, Malvern, UK) at 25  °C Hydrodynamic diameter distribution of LH2 preparations was measured before illumination with blue–green light (375 > *λ* >600 nm, 650 µmol photon s −1 m −2) with 10 mM L-histidine.

10.7717/peerj.16615/supp-10 Supplemental Information 10 Raw data exported from the Zetasizer Ultra (Malvern Panalytical, Malvern, UK) at 25  °C Hydrodynamic diameter distribution of LH2 preparations was measured after illumination with blue–green light (375 > *λ* >600 nm, 650 µmol photon s −1 m −2) in the absence of 10 mM L-histidine.

10.7717/peerj.16615/supp-11 Supplemental Information 11 Raw data exported from the SmartPave 102 rheometer (Anton Paar GmbH, Germany) Temperature dependence of viscosity of LH2 preparations solutions was measured after illumination with blue–green light (375 > *λ* >600 nm, 650 µmol photon s −1 m −2) in the absence of 10 mM L-histidine.

10.7717/peerj.16615/supp-12 Supplemental Information 12 Raw data exported from the SmartPave 102 rheometer (Anton Paar GmbH, Germany) Temperature dependence of viscosity of LH2 preparations solutions was measured before illumination with blue–green light (375 > *λ* >600 nm, 650 µmol photon s −1 m −2) in the absence of 10 mM L-histidine.

10.7717/peerj.16615/supp-13 Supplemental Information 13 Processed data about kinetics of the Spy-LHP fluorescence related to its oxidation by peroxides photoproduced before illumination of LH2 preparations from Ectothiorhodospira haloalkaliphila in the presence of 10 mM L-histidine

10.7717/peerj.16615/supp-14 Supplemental Information 14 Processed data by the “Origin 12 pro” program about kinetics of molecular oxygen photoconsumption in LH2 preparations from Ectothiorhodospira haloalkaliphila

10.7717/peerj.16615/supp-15 Supplemental Information 15 Raw data exported from Jasco FP-8300 spectrofluorimeter (JASCO Applied Sciences, Victoria, BC, Canada) at 25 °C Kinetics of the Spy-LHP fluorescence related to its oxidation by peroxides photoproduced before a illumination of LH2 preparations (83.5 nM) from Ectothiorhodospira haloalkaliphila with blue–green light (in the presence of 10 mM L-histidine.

10.7717/peerj.16615/supp-16 Supplemental Information 16 Raw data exported from Jasco FP-8300 spectrofluorimeter (JASCO Applied Sciences, Victoria, BC, Canada) at 25 °C Kinetics of the Spy-LHP fluorescence related to its oxidation by peroxides photoproduced during a 20-min illumination of LH2 preparations (83.5 nM) from Ectothiorhodospira haloalkaliphila with blue–green light (375 > *λ* >600 nm, 650 µmol photon s −1 m −2) in the presence of 10 mM L-histidine.

10.7717/peerj.16615/supp-17 Supplemental Information 17 Raw data exported from the Chirascan circular dichroism spectrometer (Applied Photophysics, UK) with a thermostabilized cell one mm cuvette, the optical density of samples at 850 nm was about 1; curve at 210 nm, upon addition of LH2 preparations (83.5 nM) from *Ectothiorhodospira haloalkaliphila*.

10.7717/peerj.16615/supp-18 Supplemental Information 18 Dependence of viscosity of LH2 preparations (83.5 nM) from Ectothiorhodospira haloalkaliphila and CD signal at 210 nm Initial data processed by the“ Origin 12 pro” program.

10.7717/peerj.16615/supp-19 Supplemental Information 19 Oxygen photoconsumption in LH2 preparations Initial data processed by the“ Origin 12 pro” program.

10.7717/peerj.16615/supp-20 Supplemental Information 20 Dynamic light scattering Initial Data

10.7717/peerj.16615/supp-21 Supplemental Information 21 Photoproduction of R-OOH in LH2 preparations Kinetics of the Spy-LHP fluorescence related to its oxidation by peroxides photoproduced during a 20-min illumination of LH2 preparations in the presence of 10 mM L-histidine.

10.7717/peerj.16615/supp-22 Supplemental Information 22 Difference (light minus dark) absorption spectrum of LH2 preparations with addition of BR before and after illumination

## Additional Information and Declarations

### Data Availability

The following information was supplied regarding data availability: The raw data are available in the Supplemental Files.
