# Supplementary figures and images for: Carotenoid-dependent singlet oxygen photogeneration in light-harvesting complex 2 of Ectothiorhodospira haloalkaliphila leads to the formation of organic hydroperoxides and damage to both pigments and protein matrix
Source: PeerJ. 2024 Jan 16;12:e16615. doi: 10.7717/peerj.16615 (PMC10798160; doi:10.7717/peerj.16615)

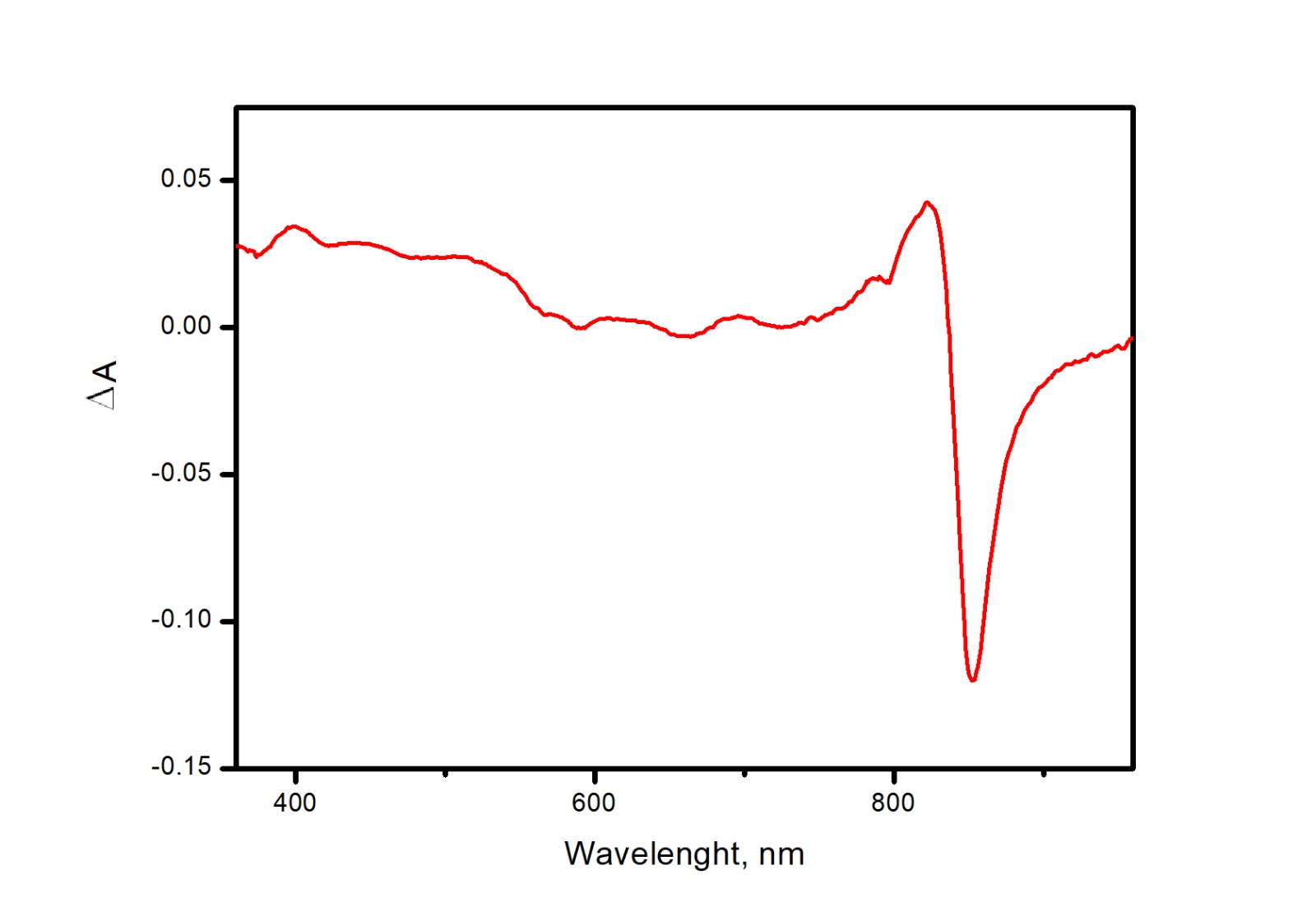

Supplement: Supplemental Information 22 [file peerj-12-16615-s022.jpeg]
